# USCA service utilization in the city of Florence (Italy) during the COVID-19 pandemic

**DOI:** 10.1093/eurpub/ckac131.075

**Published:** 2022-10-25

**Authors:** P Buscemi, B Velpini, C Cosma, C Milani, R Landi, M Innocenti, L Baggiani, M Nerattini, C Lorini, G Bonaccorsi

**Affiliations:** 1 School of Hygiene and Preventive Medicine, University of Florence, Florence, Italy; 2 Department of Health Sciences, University of Florence, Florence, Italy; 3 Azienda USL Toscana Centro, Florence, Italy; 4 Department of District Healthcare Network, Azienda USL Toscana Centro, Florence, Italy; 5 Florence Local Health District, Società della Salute di Firenze, Florence, Italy

## Abstract

**Background:**

In order to support primary care during the first pandemic wave (March 2020), the Italian Government instituted multiprofessional health teams called “USCA” (Special Continuity Care Units), which ensured continuity of care for COVID-19 patients who do not need hospitalization. The aim of our study was to compare the volumes of USCA service utilization in Florence (Tuscany, Italy) during the peak of home visits of three pandemic waves.

**Methods:**

This single-center study followed a retrospective cross-sectional design. The USCA of the Heath District of Florence served a population of 366,190 people. The following data were collected: home medical visits, nursing home (NH) visits, visits in health-care hotels. The peak periods of three epidemic waves were considered in the analyses: the second wave (23 October - 20 November 2020), the third wave (25 March - 22 April 2021), and the Omicron period (27 December 2021 - 6 February 2022). The maximum 7-day moving averages of the daily number of visits during the three periods were calculated. Relative percent differences for visits comparing the considered periods were computed.

**Results:**

Home visits during the third pandemic wave increased by 14% compared to the second wave (second wave: N = 1370, third wave: N = 1562), while a decrease was observed during the Omicron period (Omicron vs third wave: -21%; peak value: 41 vs 60). Visits in health-care hotels during the third wave doubled compared to the second wave. After the start of the COVID-19 vaccination campaign, NH visits steeply declined (third wave vs second wave: -95%; N = 323 vs 15; peak value= 14 vs 2 visits per day). During the Omicron period, NH visits increased by almost four times compared to the third wave period.

**Conclusions:**

The USCA service utilization was significant in all the analyzed periods. In a pandemic context, it is necessary to strengthen primary care services such as USCA, which have proved to respond to rapidly changing health needs.

**Key messages:**

• The USCA service is an innovative model of integrated home care that has proved to respond to rapidly changing health needs during all phases of the COVID-19 pandemic.

• The USCA service utilization was significant during all phases of the pandemic. The USCA service has introduced new ways of working and new relationships between services in primary care.

